# Acute Ethanol Administration Upregulates Synaptic α4-Subunit of Neuronal Nicotinic Acetylcholine Receptors within the Nucleus Accumbens and Amygdala

**DOI:** 10.3389/fnmol.2017.00338

**Published:** 2017-10-24

**Authors:** Josephine R. Tarren, Henry A. Lester, Arnauld Belmer, Selena E. Bartlett

**Affiliations:** ^1^Translational Research Institute, Institute for Health and Biomedical Innovation, Queensland University of Technology, Brisbane, QLD, Australia; ^2^Division of Biology and Biological Engineering, California Institute of Technology, Pasadena, CA, United States

**Keywords:** alcohol, nicotinic receptor, dopamine, nucleus accumbens, amygdala

## Abstract

Alcohol and nicotine are two of the most frequently abused drugs, with their comorbidity well described. Previous data show that chronic exposure to nicotine upregulates high-affinity nicotinic acetylcholine receptors (nAChRs) in several brain areas. Effects of ethanol on specific brain nAChR subtypes within the mesolimbic dopaminergic (DA) pathway may be a key element in the comorbidity of ethanol and nicotine. However, it is unknown how alcohol affects the abundance of these receptor proteins. In the present study, we measured the effect of acute binge ethanol on nAChR α4 subunit levels in the prefrontal cortex (PFC), nucleus accumbens (NAc), ventral tegmental area (VTA), and amygdala (Amg) by western blot analysis using a knock-in mouse line, generated with a normally functioning α4 nAChR subunit tagged with yellow fluorescent protein (YFP). We observed a robust increase in α4-YFP subunit levels in the NAc and the Amg following acute ethanol, with no changes in the PFC and VTA. To further investigate whether this upregulation was mediated by increased local mRNA transcription, we quantified mRNA levels of the *Chrna4* gene using qRT-PCR. We found no effect of ethanol on α4 mRNA expression, suggesting that the upregulation of α4 protein rather occurs post-translationally. The quantitative counting of YFP immunoreactive puncta further revealed that α4-YFP protein is upregulated in presynaptic boutons of the dopaminergic axons projecting to the shell and the core regions of the NAc as well as to the basolateral amygdala (BLA), but not to the central or lateral Amg. Together, our results demonstrate that a single exposure to binge ethanol upregulates level of synaptic α4^∗^ nAChRs in dopaminergic inputs to the NAc and BLA. This upregulation could be linked to the functional dysregulation of dopaminergic signalling observed during the development of alcohol dependence.

## Introduction

Binge drinking is the most common pattern of excessive alcohol intake and is the leading cause of death and disability globally among people between the ages of 15–49 (Lim et al., 2012). Alcohol (ethanol) in relevant concentrations functionally interferes with the release of several brain neurotransmitters, including dopamine (Bainton et al., 2000; Doyon et al., 2003), and acetylcholine, as well as their respective receptors (Larsson et al., 2005), on both activation and desensitisation. Although extensive research has examined the role of nicotinic receptors in nicotine addiction, alcohol-related changes in neuronal nAChRs involved in AUDs remain unclear. These nAChRs belong to a super-family of ligand-gated ion channel receptors, which when activated, allow for the flux of several cations. They are localised both pre- and post-synaptically and cause calcium dependent signalling cascades in many neuronal cell types (Albuquerque et al., 2009). Indeed, identification of changes in nAChRs brought about by alcohol may help to improve pharmacotherapies for alcohol dependence.

The limbic system includes several pathways between the hypothalamic-pituitary-adrenal (HPA) axis and dopaminergic systems, encompassing the PFC, NAc, VTA and the amygdala (Amg). This includes the mesocorticolimbic dopaminergic or ‘reward’ pathway, which is involved in the addiction to many drugs of abuse (Koob and Volkow, 2016). These regions are intimately connected. The NAc receives inputs from the BLA, sending outputs to the basal ganglia and ventral pallidum (Yager et al., 2015). Both receive dopaminergic innervation from the VTA. In the reward pathway (humans and rodents alike), the PFC is thought to be involved in monitoring information regarding choice, and the adjustment of behaviour in response to consequences (St Onge and Floresco, 2010), in turn, the amygdala and NAc play a role in representing the value of the reward (Mendez et al., 2013). Determining how substances of abuse such as ethanol affect nAChRs in these regions is essential to understanding how these drugs regulate reward processes and modulate decision-making and risky behaviour that further contribute to the development of alcohol dependence.

Few studies have investigated ethanol-induced changes in nicotinic receptor expression at the mRNA or protein level, and it remains an area of significance in understanding alcohol dependence. The impact of ethanol on the expression of specific nAChR subunits has mostly been investigated using heterologous systems, such as cell lines expressing nAChR subtypes. However, the mechanisms of ethanol-induced changes in nAChRs *in vivo* may differ from those in heterologous expression systems. Despite this, genetic analysis and manipulations point to the involvement of α4^∗^ nAChRs (where α4^∗^ denotes that other subunits are present in the heteropentameric nAChR protein) in alcohol addiction (Butt et al., 2003; Coon et al., 2014). The α4 subunit is the most widely expressed α subunit in the mammalian brain, including dopaminergic projection areas such as the NAc and amygdala, representing principal relay stations in the extended amygdala circuit (Exley and Cragg, 2008). Most studies agree that in the case of nicotine, alterations in presynaptic α4^∗^ nAChRs on dopaminergic neurons mediates key pathways involved in reward (Lena et al., 1999; Zhou et al., 2001; McGranahan et al., 2011), and in line with this, the partial α4β2 agonist varenicline enhances dopamine (DA) release in the NAc (Feduccia et al., 2014). Little is known about the response of this receptor subtype to ethanol, however, as in contrast to nicotine, ethanol acts primarily to potentiate the response of nAChRs to acetylcholine (ACh) rather than as a direct agonist (Zuo et al., 2002).

Although available evidence shows that transcription and upregulated protein synthesis is not directly involved in nicotine-induced increases in α4^∗^ nAChRs (Pauly et al., 1996; Miao et al., 1998; Huang and Winzer-Serhan, 2006), ethanol regulation of heteromeric nAChRs is still widely unresolved. Until recently, *in vivo* studies concerning nAChR subunits have been hindered by the lack of selectivity of receptor-specific antibodies. Indeed, several studies have concluded that under commonly used conditions, nAChR antibodies are not suitable for immunocytochemistry (Herber et al., 2004; Jones and Wonnacott, 2005; Moser et al., 2007). An advance has been the generation of transgenic knock-in mice in which the nAChR subunit is fused with YFP (Nashmi et al., 2007), and these have previously been used to successfully quantitate α4 subunit expression in the brain (Chatterjee et al., 2013; Renda and Nashmi, 2014).

Using these knock-in mice, we examine the effect of acute ethanol exposure on nAChRs containing the α4 subunit by western blot and on the gene encoding for the subunit (*Chrna4*) by real-time PCR (qPCR) in brain regions involved in the mesocortical limbic reward pathway, in particular the PFC, NAc, VTA and amygdala. We further adapted a quantitative immunohistochemistry method to determine the micron-scale localisation of any changes. Given the prominent role of dopaminergic signalling within the NAc and amygdala in the effect of alcohol, we investigated whether α4 subunit expression within the presynaptic boutons of dopaminergic fibres was modulated by binge ethanol exposure. Our results indicate that a single exposure to a sedating dose of ethanol upregulates the expression of synaptic α4^∗^-receptors in dopaminergic inputs to the NAc and BLA, directly linking alcohol to the functional dysregulations of dopaminergic signalling observed during the development of alcohol dependence.

## Materials and Methods

### Animals and Housing

The α4 nAChR YFP knock-in mice were produced by replacing exon 5 of the mus musculus *Chrna4* gene. The WT exon was replaced with an exon tagged with a YFP in the intracellular M3-M4 intracellular loop, allowing for functional fluorescently labelled α4 nAChR subunits. The tagged α4^∗^ nAChRs display similar localisation patterns in the brain and are under the control of the same promoters, enhancers, and trafficking mechanisms as their WT littermates (Nashmi et al., 2007). These mice were backcrossed on a C57BL/6J strain for ≥ 10 generations. We studied male α4-YFP mice born from heterozygous breeding pairs, with genotyping performed using PCR as previously described (Nashmi et al., 2007). All transgenic mice used were healthy and normal in their weight, appearance and showed no obvious signs of physical or neurobiological deficits. Mice used for this study were bred and housed in standard ventilated cages in climate-controlled rooms. Food, water, and environmental enrichment were available *ad libitum*. This study was carried out in accordance with the recommendations of National Health and Medical Research Council (NHMRC) guidelines to promote the well-being of animals used for scientific purposes and the Australian code for the care and use of animals for scientific purposes. The protocol was approved by the Queensland University of Technology Animal Ethics Committee and the University of Queensland Animal Ethics Committee.

### Drugs and Chemicals

For the systemic injections, ethanol (100% v/v, ThermoFisher Scientific, Hanover Park, IL, United States) was administered in a volume of 1 ml/kg of ethanol (20%, 3.6 g/kg, 20 ml/kg, i.p.) or saline (NaCl 0.9% w/v, 20 ml/kg, i.p.).

### Acute Ethanol Exposure

At the commencement of the experiment, 8–10 week old α4-YFP males were separated into treatment and control groups. Mice were weighed, and a large acute dose of 3.6 g/kg ethanol [20 mL/kg, 20% (v/v) ethanol] was administered intraperitoneally. Control was 0.9% saline administered at 20 ml/kg. The LORR and RORR were measured as previously described by Ponomarev and Crabbe (2002). Twenty-four hours post-treatment mice were deeply anaesthetised with isoflurane, and brains removed post-cervical dislocation and rapid decapitation. 1 mm coronal sections were made using an ice cold brain matrix (Australian National University) with section(s) containing brain regions of interest placed on an ice-cold platform and dissected under a microscope (Leica S6D, Buffalo Grove, IL, United States) according to visual anatomical landmarks and the atlas of Paxinos and Franklin. Brain tissue was snap frozen in RNase and DNase free tubes and stored at -80°C until processing.

### Blood Ethanol Concentration

Blood ethanol concentrations were tested at 30 min following ethanol exposure. Tail blood samples were collected in tubes containing EDTA. The assay was performed on EDTA plasma using the nicotinamide adenine dinucleotide (NAD)-ethanol dehydrogenase (ADH) spectrophotometric assay (Kristoffersen et al., 2005). All reagents used in this assay were purchased from Sigma-Aldrich (St. Louis, MO, United States). All samples and standards were run in triplicate, with BECs measured against a standard calibration curve.

### Measurement of α4 Subunit nAChR Levels by Western Blot

Appropriate volumes of cold lysis buffer – phosphate buffered saline (PBS) containing 0.1% Triton-X and protease inhibitor (Thermo Fisher Scientific, Crystal Lake, IL, United States) were added to samples on ice, and homogenised using 0.5 mm glass beads. Samples were centrifuged, with supernatant plus appropriate standards (Albumin Standard, Thermo Fisher Scientific, Crystal Lake, IL, United States) loaded onto a 96-well plate in triplicate with Bradford reagent (Bio-Rad, CA, cat). Absorbance was measured at 595 nm and protein level determined from the standard curve. Remaining supernatant was prepared for electrophoresis to a concentration of 30 μg/lane with laemmli sample buffer (Bio-Rad, United States) containing dithiothreitol (DTT) and incubated at 37°C.

Proteins were separated using sodium dodecyl sulphate polyacrylamide gel electrophoresis (SDS–PAGE) using 4-20% pre-cast Tris-glycine gels (Bio-Rad, United States) and blotted to a polyvinylidene fluoride (PVDF) membrane in transfer buffer containing 20% methanol. Protein migration was assessed relative to the migration of Precision Plus Protein^TM^ dual colour standard (10–250 kDa). Membranes were washed (1 × 20 min, 2 × 10 min) and then blocked using 5% dry milk solution in 0.05% Tween 20 in PBS. Post-blocking, blots were probed overnight with monoclonal mouse anti-GAPDH antibody (1:15000, Thermo Fisher Scientific, Crystal Lake, IL, United States), mouse monoclonal anti-GFP antibody to detect YFP (1:500, Cell Signaling Technologies, Danvers, MA, United States) and mouse anti-TH (Millipore #MAB318, 1:10000) in the blocking solution. Washing was repeated, and membranes developed with donkey anti-mouse IgG (H&L) tagged with DyLight^TM^ 800 (1:1000, Rockland, PA, United States) for 1 h at RT in 0.05% Tween 20/PBS. Membranes were washed, then dried out at 4°C using desiccant beads. Bands of interest were visualised using the Odyssey infrared imager (LI-COR Biosciences, Lincoln, NE, United States) and band densities (K counts) quantified using the Odyssey 2.0.40 software (LI-COR).

### Measurement of α4 Subunit nAChR Levels by Quantitative Immunohistochemistry

#### Histology

Twenty-four hours after ethanol injection, animals were transcardially perfused with 4% paraformaldehyde (PFA) prior to decapitation. Brains were harvested and post-fixed overnight at 4°C. Thirty-micron thick coronal vibratome sections were collected and immersed in ice-cold 10% methanol-PBS solution for 5 min. After three washes in citrate buffer solution (10 mM citrate buffer, 0.05% Tween 20, pH 6.0) at room temperature, sections were incubated in a 37°C pre-heated citrate buffer solution and placed at 80°C for 20 min for antigen retrieval, rinsed 3 times in PBS and incubated overnight in blocking solution (3% bovine serum albumin BSA, 0.3% Triton and 0.05% Tween 20).

#### Immunohistochemistry

Sections containing the NAc (Bregma 1.70 to 0.90 mm) or the amygdala (Bregma -1.40 to -2.00 mm) were incubated overnight at 4°C with primary antibodies diluted in the blocking solution: rabbit anti-GFP (Abcam #290, 1:5000) and mouse anti-TH (Millipore #MAB318, 1:500) and, after three washes in the blocking solution, incubated with secondary antibodies diluted in the blocking solution overnight at 4°C: donkey anti-rabbit-Alexa 488 and donkey anti-mouse-Alexa 647 (Thermofisher Scientific, 1:500). Sections were mounted in Prolong Gold antifade mountant (Thermofisher Scientific). For synaptic α4-YFP puncta detection, sections were incubated overnight at room temperature with mouse anti-synaptophysin antibody (Sigma #S5768, 1:200), followed by 4h incubation with goat anti-mouse monovalent Fab-Alexa 647 (Jackson #115607003, 1:500). Free mouse epitopes were blocked with anti-mouse unconjugated monovalent Fab antibodies (Abcam #ab6668, 1:100) overnight at 4°C. GFP and TH immunolabelling were conducted as above with goat-raised secondary antibodies (Thermofisher Scientific, 1:500).

#### Imaging and Analysis

**FIGURE 1:**
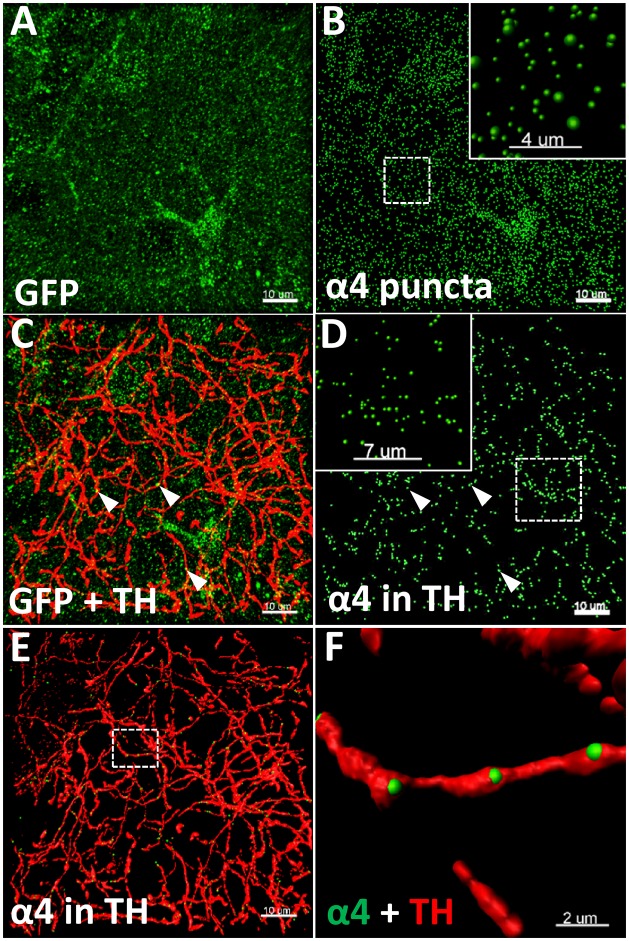
Illustration of the quantitative immunohistochemistry method used to determine α4YFP levels. **(A–F)** Representative images taken from the CeA of saline treated mice. **(A)** Micrographs representing the immunolabelling of α4-YFP (GFP, green; scale bar: 10 μm). **(B)** Reconstruction of α4 puncta from α4-YFP labelling (scale bar: 10 μm). Top right box represents a higher magnification of the dashed square (scale bar: 4 μm). **(C)** Double immunolabelling of α4-YFP puncta (GFP, green) and dopaminergic fibres (TH, red; scale bar: 10 μm). **(D)** Isolation of α4-YFP puncta (GFP, green) located within dopaminergic fibres (scale bar: 10 μm). Top left box represents a higher magnification of the dashed square (scale bar: 7 μm). In **(C,D)**, arrow heads show examples of α4-YFP puncta in TH fibres. **(E)** 3D-reconstruction of α4-YFP puncta (GFP, green) within dopaminergic fibres and dopaminergic fibres (TH, red; scale bar: 10 μm). **(F)** Higher magnification of the dashed square in **E**, showing α4YFP puncta within dopaminergic fibres (GFP, green), and dopaminergic fibres (TH, red; scale bar: 2 μm).

Three sections per animal (*n* = 5–6 animals/group, 15–18 images/brain region/group) were imaged on an Olympus FV1200 confocal microscope using a 60X oil-immersion objective (NA 1.35) with 2.5 × zoom, with a *Z*-axis step of 0.3 μm. Images were deconvolved using Huygens Professional v16.10 (Scientific Volume Imaging) with 100 iterations, quality threshold at 0.001, signal to noise ratio at 15 for the three channels. Images were subsequently analysed in Imaris 8.2.1 (Bitplane), as previously described (Belmer et al., 2017). Total YFP punctate fluorescence (α4-YFP) (**Figure 1A**) was quantified using the spot detection function of Imaris, with a spot diameter of 0.4 μm (**Figure 1B**). TH immunolabelled fibres (**Figure 1C**) were reconstructed in 3D using the surface rendering function (**Figures 1E,F**). The YFP punctate fluorescence within TH reconstructed fibres (α4-YFP^TH+^), was isolated using the masking function and quantified using the spot detection function (**Figure 1D**). The spot colocation function was used to identify and quantify the synaptic YFP punctate fluorescence co-localised with synaptophysin puncta within TH reconstructed fibres (α4-YFP^SY N/TH+^; *n* = 5–6 animals/group, 15 images/brain region/group). The density of α4-YFP puncta or TH immunolabelled fibres was calculated per μm^3^ of tissue, and the density of α4-YFP^TH+^ puncta (**Figures 1E,F**) calculated per μm^3^ of TH immunoreactive fibres. The proportion of α4-YFP^TH+^ puncta (α4-YFP^SY N/TH+^) was calculated in percent of total α4-YFP^TH+^.

### Measurement of *Chrna4* mRNA Level Using qRT-PCR

Prior to RNA extraction, RNAlater^®^ was added to the samples at 10× the volume of tissue and incubated at -20°C for a minimum of 16 h. Total RNA extraction was then performed using the RNeasy^®^ micro kit (Qiagen, Hilden, GER) as per manufacturer’s instructions. RNA concentration and purity were assessed using the NanoDrop^TM^ 1000 UV-Vis spectrophotometer. For removal of contaminant DNA, 500 ng of total RNA was treated with DNase I, amplification grade (1 U; 10 μL total volume). First strand synthesis was carried out using the SuperScript^®^ III First-Strand Synthesis System for qRT-PCR as per the manufacturer’s instructions. For qRT-PCR, cDNA (equivalent of 500 ng RNA starting material) was diluted 1/10 in nuclease-free water. To 2 μL diluted cDNA was added SYBR^®^ Green Real-Time PCR Master Mix (1 × final concentration), and forward and reverse primer (10 nM each) to a final volume of 10 μL. Primers for GAPDH and HPRT were used as normalisation controls. Primer sequences are detailed in **Table 1**. Reactions were conducted in the ViiA^TM^ 7 (Applied Biosystem, Foster, CA, United States) Real-Time PCR System with the following series of thermocycling steps: 95°C, 10 min; 40× cycles of 95°C, 15 s; and 63°C, 10 min. After each PCR reaction, the specificity of the amplification was evaluated by a melt curve analysis, and plates controlled for genomic DNA contamination using controls omitting the cDNA template and the reverse transcriptase step, respectively. Primer dimers were ruled out using end point PCR, with band specificity checked by agarose gel electrophoresis. Amplification of GAPDH was measured in some –RT controls, this was considered negligible at <0.01% of cDNA amplified. All reagents used in this assay unless specified were purchased from Thermo Fisher Scientific (Crystal Lake, IL, United States). For gene primer sets (from Invitrogen, United States) see **Table 1**. Correction for sample-sample variation was done by simultaneously amplifying both GAPDH and HPRT as a reference. Specific PCR amplification efficiencies for each gene were generated individually for each brain region tested using a 7 point Ct (cycle threshold) slope method, with calibration curves covering a 3-log range with twofold serial dilutions of cDNA transcript. The Ct values of each sample were normalised with the mean Ct value for the internal reference genes and were corrected for the PCR efficiency of each assay.

**Table 1:** Nucleotide sequences of PCR and qRT-PCR primers.

Accession No.	Target	Forward	Reverse	Amplicon
**Genotyping**				
	α4XFP2	F, 5′-CAACCGCATGGACAC AGCAGTCGAGAC-3′ M, 5′-GCACAAGCTGGAGTA CAACTACAACAGC-3′	5′-CTCAGTCAGGGAA GCAGCTCCATCTTG-3′	542
**qRT-PCR**				
NM_015730.5	*Chrna4*	5′-ACTTCTCGGTGAAG GAGGACT-3′	5′-GCCCAGAAGGCAG ACAATGAT-3′	89
NM_001289726.1	GAPDH (Beckley et al., 2016)	5′-CGACTTCAACAGCA ACTCCCACTCTTCC-3′	5′-TGGGTGGTCCAGGG TTTCTTACTCCTT-3′	175
NM_013556.2	HPRT	5′-TCCCAGCGTCGTG ATTAGCGATGA-3′	5′-AATGTGATGGCCTC CCATCTCCTTCATGACAT-3′	172


### Statistics

Statistical analyses were carried out using GraphPad Prism 7 (Graph Pad Software Co., San Diego, CA, United States). Statistical comparisons for western blot analysis were performed using a two-tailed unpaired *t*-test, with Holm-Sidak’s correction for multiple comparison, and two-way ANOVA with Sidak’s multiple comparison (without including the PFC due to large standard deviation of the dataset, see details in the results section). A *p*-value < 0.05 was considered significant, with all values expressed as the mean ± SEM. Real-time qPCR data were analyzed using the 2^ΔΔCt^ method (Pfaffl, 2001). Each data point represents an average of three technical replicates, with two-way ANOVA analysis followed by Sidak’s comparison, performed with linear delta Ct values prior to transformation. One sample showed marked degradation across all primer sets and was excluded from analysis. A *p*-value < 0.05 was considered significant, with biological significance achieved at a twofold change in expression. The volumetric density of α4-YFP puncta, TH fibres, α4-YFP^TH+^ and α4-YFP^SY N/TH+^ puncta were analysed by two-tailed unpaired *t*-test or two-way ANOVA followed by Sidak’s multiple comparison test. A *p*-value < 0.05 was considered significant, with all values expressed as the mean ± SEM.

## Results

**FIGURE 2:**
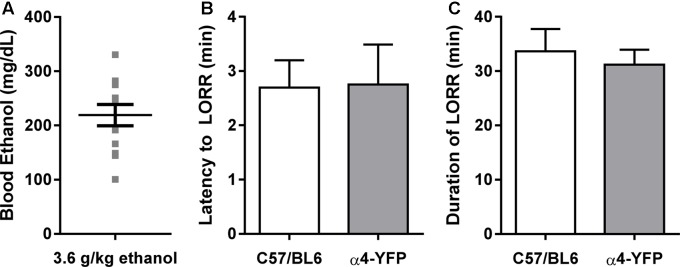
Sensitivity to ethanol in α4-YFP-KI mice. **(A)** Blood ethanol concentrations following a single sedating dose of ethanol. Data are presented as mean ethanol concentration in mg per dL ± SEM. **(B,C)** The latency to, and duration of the sedative effects of ethanol show no differences between C57Bl6 and α4-YFP mice. Data are presented as mean latency to LORR in min ± SEM **(B)**, and duration of LORR in min ± SEM **(C)**; *p* = 0.95 and *p* = 0.63, respectively.

We examined the effect of an acute large dose of ethanol (3.6 g/kg, i.p.) or saline (20 ml/kg, i.p) on the animal’s LORR and RORR in order to measure the sedative effect of ethanol in each animal. Latency to and duration of LORR was analogous to that of similarly treated C57BL/6 mice (**Figures 2B,C**). In agreement with previous studies, the α4-YFP mice, showed no physiological alterations compared to their WT littermates (Nashmi et al., 2003, 2007; Santos et al., 2013). BECs were also measured to confirm treatment (**Figure 2A**).

### Acute Exposure to Ethanol Up-regulates α4 Subunit in the NAc and Amygdala

**FIGURE 3:**
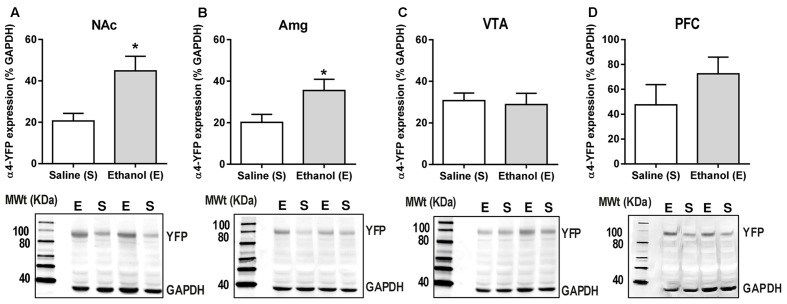
Acute ethanol exposure significantly increases levels of α4 subunit in the nucleus accumbens and amygdala but not in the ventral tegmental area and prefrontal cortex. **(A,B)** The level of YFP was quantified 20–22 h post-ethanol exposure using western Blot analysis. Data are presented as mean k counts for YFP expressed as a percentage of GAPDH k counts ± SEM (two-tailed unpaired *t*-test with Bonferroni-Sidak’s correction for multiple analysis **(A)** NAc ^∗^*p* = 0.034, *n* = 8; **(B)** Amg ^∗^*p* = 0.037, *n* = 7–8; **(C)** VTA: *p* = 0.99, *n* = 4; and **(D)** PFC: *p* = 0.69, *n* = 7–8). Representative western blots showing the level of YFP and GAPDH expression of both saline (S) and ethanol (E) administered α4-YFP mice are shown for each brain region below the corresponding graphs.

Levels of α4-YFP protein in the NAc, amygdala, VTA and PFC were measured 24 h post-ethanol or saline treatment using a homogenate western blot procedure. Band densities were measured as integrated intensity (k counts) and quantified as a percent (%) of GAPDH. Ethanol administration significantly increased α4YFP protein levels in both the NAc (44.84 ± 7.03 k counts, *n* = 8, **Figure 3A**) and the amygdala (35.49 ± 5.43 k counts, *n* = 8, **Figure 3B**) compared to the control mice (20.62 ± 3.70 k counts, *n* = 8 and 20.19 ± 3.14 k counts, *n* = 7, respectively) using a two-tailed unpaired *t*-test with Bonferroni-Sidak’s correction for multiple comparison, ^∗^*p* = 0.034 (NAc) and ^∗^*p* = 0.037 (Amg). Interestingly, no effect was seen in the VTA (28.86 ± 5.398 k counts, *n* = 4, **Figure 3C**), or the PFC (72.46 ± 13.45 k counts, *n* = 8, **Figure 3D**), compared to the control mice (30.73 ± 3.627, *n* = 4 and 47.51 ± 16.24, *n* = 7, respectively), suggesting that increases in α4 protein levels may only occur in specific brain regions. Using two-way ANOVA, we observed significant effect of ethanol treatment *F*(1,33) = 9.69, ^∗∗^*p* = 0.004, with Sidak’s comparison showing a significant effect of ethanol treatment in the NAc (^∗∗^*p* = 0.003) and the Amg (^∗^*p* = 0.027), with no effect in the VTA (*p* = 0.99). PFC was not included in the analysis due to a large standard deviation, compared to NAc, Amg and VTA. This large standard deviation is likely due to a high heterogeneity of both the cholinergic/dopaminergic neurons within the different layers of the medial vs. infralimbic prefrontal cortices (Zhang et al., 2010). Treatment with ethanol did not affect the expression of TH in either the NAc or amygdala (**Supplementary Figures S1a,b**).

### Effect of Acute Ethanol Administration on the Expression of α4 Subunits within the Mesocortical Limbic System Is Not Mediated by Changes in *Chrna4* mRNA Level

**FIGURE 4:**
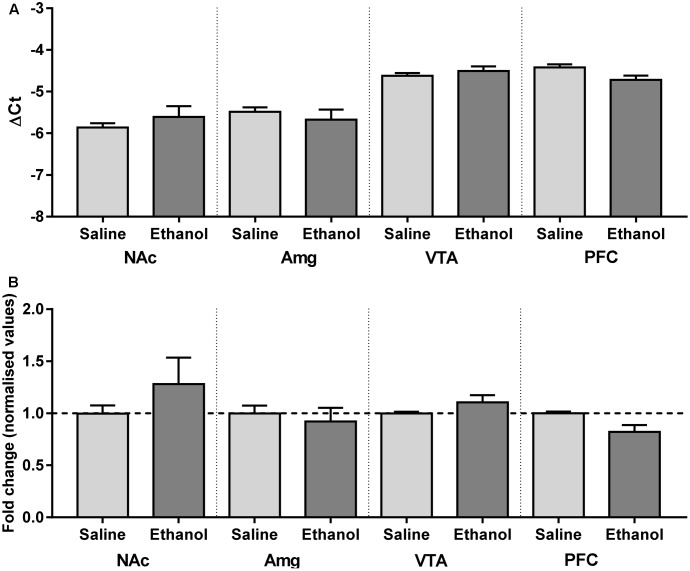
*Chrna4* levels following acute ethanol exposure are not altered in the nucleus accumbens, amygdala, ventral tegmental area and prefrontal cortex. **(A)** Change in Ct was normalised against controls GAPDH and HPRT and analysed using two-way ANOVA followed by Sidak’s *post hoc* analysis on linear delta Ct values prior to transformation; see **Table 2** for detailed statistical analysis. **(B)** Values were then corrected for the individual PCR efficiency of each brain region using a 7-point curve represented as a fold-change from control (saline-treated) mice values (1.0). No biological significance was seen, with no brain region exhibiting a twofold change in expression compared to control. Data are presented as mean ± SEM.

Although changes in mRNA expression do not accompany increased expression of heteromeric nAChRs caused by nicotine, ethanol has a quite different pharmacology, and alters the mRNA profiles of similar ligand-gated receptors, such as NMDA and GABAA receptor subunit genes (Mhatre and Ticku, 1992; Xiang et al., 2015). To test whether increases in receptor protein are partly due to increases in gene expression, mRNA primers were generated to detect α4 subunit mRNAs in brain tissue from male α4-YFP mice. Regions with high levels of α4-YFP mRNA expressions were also those with high levels of baseline expression in western blot analysis. To determine whether the ethanol-induced up-regulation of α4-YFP levels represented an increase in production in mRNA, brains were processed for quantitative analysis by reverse transcriptase PCR. Compared to the brain region-specific controls, mRNA expression was not significantly altered 24 h after ethanol treatment (two-way ANOVA, [alcohol treatment]: *F*_(1,36)_ = 0.1197, *p* = 0.73; followed by Sidak’s *post ho*c comparison: *p* > 0.05, **Figure 4** and **Table 2**).

**Table 2:** Region specific mRNA ratios.

Brain region	Efficiency		ΔCt Control (n)	ΔCt Ethanol (n)	Sidak’s test	2^ΔΔ*C*t^
Amygdala	*Chrna4* GAPDH HPRT	2.10 2.03 1.94	-5.487 ± 0.11 (6)	-5.675 ± 0.24 (6)	NS: *p* = 0.99	0.93 ± 0.13 (*p* = 0.98)
Nucleus accumbens	*Chrna4* GAPDH HPRT	2.20 2.09 1.93	-5.860 ± 0.10 (6)	-5.608 ± 0.26 (6)	NS: *p* = 0.35	1.35 ± 0.31 (*p* = 0.35)
Ventral tegmental area	*Chrna4* GAPDH HPRT	2.29 2.36 2.24	-4.620 ± 0.06 (4)	-4.508 ± 0.11 (4)	NS: *p* = 0.99	1.10 ± 0.06 (*p* > 0.99)
Prefrontal cortex	*Chrna4* GAPDH HPRT	2.09 2.08 1.97	-4.651 ± 0.10 (5)	-4.418 ± 0.08 (5)	NS: *p* = 0.88	0.89 ± 0.09 (*p* = 0.94)


### Acute Ethanol Administration Upregulates the Expression of α4 Subunits in Dopaminergic Fibres within the NAc Core and Shell and the BLA

**FIGURE 5:**
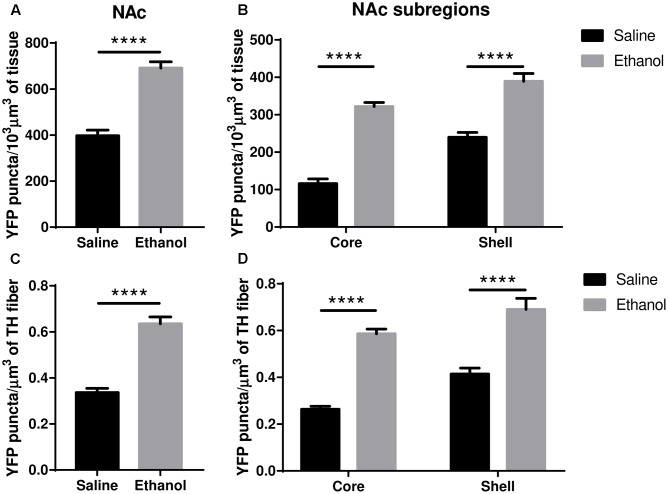
Acute ethanol exposure significantly increases levels of α4 subunit in the nucleus accumbens shell and core. **(A,B)** Effect of acute ethanol on the volumetric density of YFP puncta in the NAc **(A)** or the core and shell **(B)** subregions. Data are presented as mean puncta density per 10^3^ μm^3^ of tissue ± SEM. (**A**: two-tailed unpaired *t*-test, ^∗∗∗∗^*p* < 0.0001, compared to saline; **B**: Two-way ANOVA, followed by Sidak *post hoc* analysis, ^∗∗∗∗^*p* < 0.0001, compared to saline). **(C,D)** Effect of acute ethanol on the volumetric density of YFP puncta within TH immunoreactive fibres in the NAc **(C)**, or in the core and shell **(D)** subregions. Data are expressed as mean puncta density per μm^3^ of TH immunoreactive fibres ± SEM. (**C**: two-tailed unpaired *t*-test, ^∗∗∗∗^*p* < 0.0001, compared to saline; **D**: Two-way ANOVA, followed by Sidak *post hoc* analysis, ^∗∗∗∗^*p* < 0.0001, compared to saline).

The upregulation of nAChRs by nicotine is selective in several respects but usually involves α4^∗^ nAChRs, and the α4^∗^ nAChRs of dopaminergic nerves are among those upregulated (see “Discussion”). We, therefore, examined ethanol-induced changes in α4 subunit levels within dopaminergic (TH immunoreactive) inputs to the core/shell of the NAc, ad BLA, LA and CeA of the amygdala. In the NAc (core + shell), we observed a significant increase in the total volumetric density of α4-YFP puncta (**Figure 5A**, *t*-test, ^∗∗∗∗^*p* < 0.0001) following acute ethanol administration. This was driven by an increase in the density of α4-YFP puncta both in the core and the shell (**Figure 5B**, two-way ANOVA: [ethanol treatment]: *F*_(1,28)_ = 33.1, ^∗∗∗∗^*p* < 0.0001, with Sidak’s multiple comparison revealing a significant effect of ethanol treatment in both the NAc core and shell: ^∗∗∗∗^*p* < 0.0001). Interestingly, we observed a similar increase in the volumetric density of α4-YFP puncta within TH immunoreactive fibres in the NAc (core + shell) following acute ethanol administration (**Figure 5C**, *t*-test, ^∗∗∗∗^*p* < 0.0001). Similarly, this was the result of an increased density of α4-YFP puncta within TH immunoreactive fibres in both the core and the shell (**Figure 5D**, two-way ANOVA: [ethanol treatment]: *F*(_1,62)_ = 91.267, ^∗∗∗∗^*p* < 0.0001, with Sidak’s multiple comparison revealing a significant effect of ethanol treatment in both the NAc core and shell, ^∗∗∗∗^*p* < 0.0001). We did not observe any change in the overall density of TH immunoreactive fibres in the NAc (saline: 484.5 ± 25.0 vs. ethanol: 497.6 ± 12.4 mm^3^/10^3^mm^3^ of tissue, *t*-test, *p* = 0.63, **Supplementary Figure S1c**).

**FIGURE 6:**
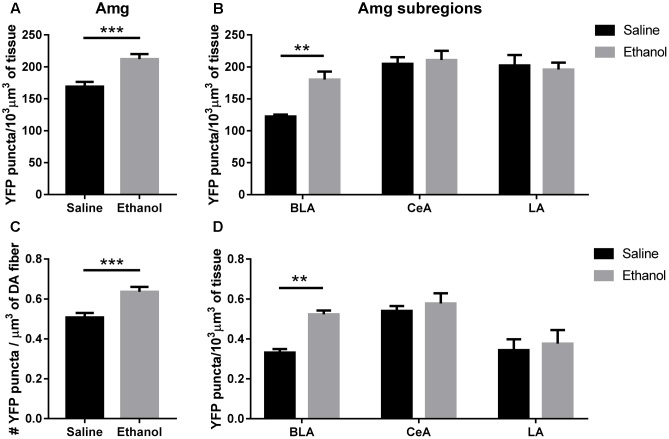
Acute ethanol exposure significantly increases levels of α4 subunit in the basolateral amygdala but not the central or lateral amygdala. **(A,B)** Effect of acute ethanol on the volumetric density of YFP puncta in the amygdala **(A)**, or the BLA, CeA and LA **(B)** subregions. Data are presented as mean puncta density per 10^3^ μm^3^ of tissue ± SEM. (**A**: two-tailed unpaired *t*-test, ^∗∗∗^*p* < 0.001, compared to saline; **B**: Two-way ANOVA, followed by Sidak *post hoc* analysis, ^∗∗^*p* < 0.01, compared to saline). **(C,D)** Effect of acute ethanol on the volumetric density of YFP puncta within TH immunoreactive fibres in the amygdala **(C)**, or the BLA, CeA and LA **(D)** subregions. Data are expressed as mean puncta density per μm^3^ of TH immunoreactive fibres ± SEM. (**C**: two-tailed unpaired *t*-test, ^∗∗∗^*p* < 0.001, compared to saline; **D**: Two-way ANOVA, followed by Sidak *post hoc* analysis, ^∗∗^*p* < 0.01, compared to saline).

The total volumetric density of α4-YFP puncta was also increased in the amygdala (BLA + CeA + LA) (**Figure 6A**; *t*-test, ^∗∗∗^*p* = 0.0007), and this was mostly driven by an increase in α4-YFP puncta density in the BLA (**Figure 6B**, two-way ANOVA, [ethanol treatment]: *F*_(1,90)_ = 3.655, *p* = 0.0591 with Sidak’s multiple comparison revealing a significant effect of ethanol treatment in the BLA (^∗∗^*p* < 0.002), with no significant changes in the CeA (*p* = 0.98) or the LA (*p* = 0.97). As a result, the volumetric density of α4-YFP puncta within TH immunoreactive fibres was also increased in the overall amygdala (**Figure 6C**, *t*-test, ^∗∗∗^*p* = 0.007), resulting principally from an increased α4-YFP puncta density within TH immunoreactive axons (two-way ANOVA, [ethanol treatment]: *F*_(1,90)_ = 5.631, ^∗^*p* = 0.0198) located in the BLA (Sidak’s post-test: ^∗∗^*p* = 0.0059), with no change in the LA (*p* = 0.93) and the CeA (*p* = 0.93) (**Figure 6D**). The density of TH immunoreactive axons was unchanged in the amygdala (saline: 219.4 ± 12.56 vs. ethanol: 219.5 ± 20.33 mm^3^/10^3^mm^3^ of tissue, *t*-test: *p* = 0.10, **Supplementary Figure S1d**). As the anti-TH antibody that we used predominantly labels dopaminergic neurons (**Supplementary Figure S2a**) and terminals (**Supplementary Figure S2c**), compared to noradrenergic neurons (**Supplementary Figure S2b**) and terminals (**Supplementary Figure S2d**), the reconstructed TH immunoreactive fibres are likely dopaminergic.

### Acute Ethanol Administration Increases the Distribution of α4 Subunits in Synaptophysin-Immunoreactive Presynaptic Boutons within Dopaminergic Fibres in the NAc Core, and Shell and BLA

**FIGURE 7:**
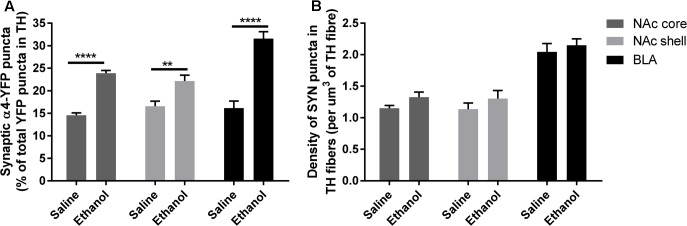
Acute ethanol exposure significantly increases the proportion of synaptic α4-YFP puncta co-localised with the marker of presynaptic boutons, synaptophysin, within TH-imunoreactive terminals in the nucleus accumbens and basolateral amygdala. **(A)** Effect of acute ethanol on the co-localisation of YFP puncta with synaptophysin puncta within TH immunoreactive axons in the NAc core, shell and BLA. Data are presented as a proportion of YFP puncta co-localised with synaptophysin (SYN) puncta in TH fibres, as a percent of total YFP puncta in TH fibres ± SEM (Two-way ANOVA, followed by Sidak *post hoc* analysis, ^∗∗∗^*p* = 0.0003; ^∗∗∗∗^*p* < 0.0001; compared to saline). **(B)** Effect of acute ethanol on the density of synaptophysin puncta within TH-immunoreactive fibres. Data are presented as mean density of SYN puncta per μm^3^ of TH reconstructed fibres ± SEM (Two-way ANOVA, followed by Sidak *post hoc* analysis, non-significant).

**FIGURE 8:**
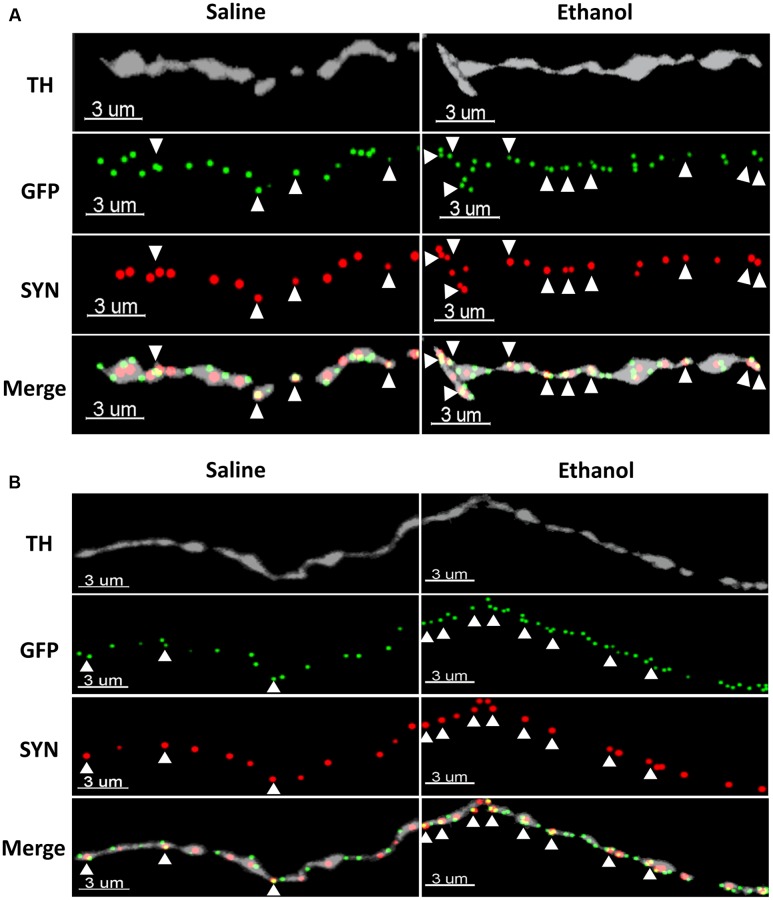
Representative micrographs showing the ethanol-induced upregulation of α4-YFP puncta within the synaptic boutons of dopaminergic terminals in the NAc **(A)** and the BLA **(B)**. The top horizontal panel shows a TH immunolabelled axon (grey), followed by the anti-GFP (YFP) staining of α4 receptor puncta (green) and synaptophysin puncta (red). The bottom horizontal panel shows the merge of upper panels. White arrowheads show synaptophysin puncta co-localised with one or more α4 receptor puncta (yellow). Scale bar: 3 μm.

To assess if the upregulation of α4 subunits could have functional consequences, we determined whether it occurs in presynaptic boutons within TH axons. For this, we quantified the proportion of total α4-YFP puncta co-localised with the marker of presynaptic boutons synaptophysin, in TH-immunoreactive axons. We observed a significant effect of [ethanol treatment]: *F*_(1,24)_ = 108.3, ^∗∗∗∗^*p* < 0.001, with Sidak’s *post hoc* comparison revealing an increased proportion of synaptic α4-YFP puncta (α4-YFP^SY N/TH^) in all the subregions analysed (NAc core, NAc shell and BLA) following acute alcohol treatment (**Figure 7A**, ^∗∗∗^*p* = 0.0003; ^∗∗∗∗^*p* < 0.0001). This effect was independent of changes in the density of synaptophysin boutons within TH-immunoreactive axons (SYN^TH^) as there was no significant effect within [ethanol treatment] factor: *F*_(1,24)_ = 3.16, *p* = 0.081. Sidak’s *post hoc* comparison revealed no significant effect of ethanol on the density of SYN^TH^ puncta in each brain region (**Figure 7B**, NAc core: *p* = 0.56; NAc shell: *p* = 0.59 and BLA: *p* = 0.86). Representative micrographs of the effect of ethanol on the synaptic distribution of α4-YFP^SY N/TH^ puncta in the NAc and BLA are provided (**Figures 8A,B**, respectively), showing that alcohol treatment increases the proportion of α4-YFP^SY N/TH^ puncta [NAc 16% (saline) vs. 25% (ethanol), **Figure 8A**; BLA 17% (saline) to 34% (ethanol), **Figure 8B**]. Consequently, the proportion of α4-YFP^TH^ puncta not co-localised with SYN puncta was decreased both in the NAc [84% (saline) vs. 75% (ethanol)] and the BLA [83% (saline) vs. 66% (ethanol)].

## Discussion

In the past, studies examining changes in nAChRs have used receptor-specific antibodies; however, such analyses raise questions due to lack of selectivity of the antibodies for the desired subunit. In this study, we have used α4-YFP knock-in mice to evaluate whether ethanol exposure affects the levels of α4 nAChR subunit protein and/or the Chrna4 mRNA that encodes this subunit. This mouse line displays normal subcellular localisation, ACh-induced currents and ACh-induced Ca^2+^ fluxes (Nashmi et al., 2007). We have also previously used these mice to identify reduced α4^∗^ nAChR levels in α5 knock-out mice using western blot (Chatterjee et al., 2013), with Renda and Nashmi (2014) only recently using the same strain to characterise an upregulation of α4^∗^ nAChRs after chronic nicotine pre-treatment using qualitative measurements of fluorescence intensity. In the present study, we have adapted a quantitative approach (Belmer et al., 2017) to demonstrate that synaptic α4^∗^ nAChRs, in specifically dopaminergic fibres within the NAc and BLA, are upregulated just hours after a single ‘binge’ episode of alcohol administration. For this, we colocalised the α4^∗^ nAChRs with the presynaptic bouton marker synaptophysin in dopaminergic axons using a high-resolution confocal laser scanning microscopy approach. Although synaptophysin is a one of the most widely used protein markers of functional synapses and synaptic plasticity in the brain, this approach is not resolutive enough to conclude on the subsynaptic localisation of α4^∗^ nAChRs and whether they are located at the synaptic membrane (functional), or located within the submembrane cytoskeleton (inactive). Pharmacology and physiology experiments will therefore be needed to determine whether the alcohol-upregulated α4^∗^ nAChRs are pharmacology active and functional.

ACh influences numerous physiological and pathological processes in the CNS via activation of nAChRs (Albuquerque et al., 2009) that modulate neurotransmitter release (MacDermott et al., 1999). The activation of DA receptors can increase acetylcholine release, (DeBoer et al., 1996; Nicola et al., 2000), and nAChRs on dopamine terminals play a key role in facilitating endogenous acetylcholine’s ability to trigger synaptic dopamine release (Marubio et al., 2003; Mao et al., 2011; Cachope et al., 2012; Threlfell et al., 2012). This circuit is potentiated by ethanol, and reduced in mice lacking the α4 subunit (Liu et al., 2013). Interestingly, the increases in α4^∗^ nAChRs seen in the amygdala were comprised solely of changes within the BLA. St Onge et al. (2012) previously revealed that disruption in communication between the BLA and NAc biassed choice toward larger, uncertain rewards on a probabilistic discounting task in rodents. Importantly, release of dopamine into the BLA may be distinctly involved in the response to both rewarding and threatening stimuli (Phillips et al., 2003), and changes in synaptic nAChRs on dopaminergic terminals may influence further responses, with damaged dopamine signalling also linked to several neuropsychiatric disorders (Ledonne and Mercuri, 2017). Many previous studies show that direct interaction of BLA activity and NAc dopamine is essential to conditioned reward associations (Ambroggi et al., 2008; Jones et al., 2010) and likely confers the motivational value of alcohol-related stimuli (Ghods-Sharifi et al., 2009). Interestingly, while most of the increase occurred in dopaminergic neurons, a small subset of receptors was upregulated on non-TH^+^ fibres. These may comprise nAChRs on GABAergic interneurons or serotonergic terminals, with conflicting reports of presynaptic nAChR modulation of ACh release from cholinergic axons (Schwartz et al., 1984; Pradhan et al., 2002; Quik and Wonnacott, 2011).

Current research indicates that nAChR subunit genes are potential contributors to the development of both alcoholism and tobacco abuse (Schlaepfer et al., 2008; Hoft et al., 2009a,b; Zuo et al., 2016). Also, SNP analysis of CHRNA4 by Tritto et al. (2001) and Feng et al. (2004), demonstrated that two SNPs are associated with a protective effect against nicotine and alcohol addiction in humans and mice (Tritto et al., 2001; Feng et al., 2004) when exposed chronically. Combined with the results reported here, it is likely that while polymorphisms of the CHRNA4 gene play a role in altering the sensitivity of the subunit to ethanol (Butt et al., 2003; Coon et al., 2014), acute doses of ethanol do not influence transcription.

Previous studies in this area are few and have produced mixed results. An early study on chronic ethanol exposure showed an increase in α4 mRNA levels of 22% after 4 days of direct application of ethanol to neuronal cell culture (Gorbounova et al., 1998). A recent study, and the only found addressing acute ethanol exposure reported no change in α4 mRNA expression after 24 h of application in foetal neural progenitor cells (Balaraman et al., 2012). Interestingly, the same study found a decrease in nAChR subunit mRNAs in a longer term of application (5 days). An *in vivo* study on nicotine reported a drop in the level of CHRNA4 gene expression in the VTA and an increased expression in the NAc in rat pups after 4 weeks of gestational exposure (Chen et al., 2005). It could be suggested that α4 mRNA levels may be altered only in long-term ethanol consumption, and possibly be limited to distinct developmental periods such as prenatal. As for protein expression, a previous ligand binding study reported an increase in α4β2 nAChRs in M10 cells exposed to ethanol after 48 h, which remained elevated for as long as 6 days after removal of the drug (Dohrman and Reiter, 2003). While not only being a study using cell culture, the cells specifically expressed only α4β2 nAChRs and was a purely *in vitro* model. In the mammalian brain, α4 nAChR subunits may also form receptors with the α5 nAChR subunit, which plays a distinct role in driving the expression of α4^∗^ receptors (Chatterjee et al., 2013), making the *in vivo* study of ethanol on α4^∗^ receptors valuable.

Studies that have focused on the function of nAChRs have demonstrated that deleting the α4 nAChR subunit caused increased sensitivity to nicotine-induced locomotor depression and decreased sensitivity to anxiolytic effects (McGranahan et al., 2011), suggesting an involvement of the amygdala. In addition, other groups showed that α4-knockout (KO) mice did not systemically self-administer nicotine and displayed a lack of nicotine-induced dopamine level increase seen in WT mice exposed to nicotine (Marubio et al., 2003; Pons et al., 2008). Similar observations were made in relation to alcohol addiction, as ethanol-induced place preference was absent in α4KO mice and was increased in mice with a single point mutation producing hypersensitive α4^∗^ nAChRs (Liu et al., 2013). This suggests that the α4 subunit is necessary for the development of alcohol and nicotine dependence and increased levels of the subunit will affect susceptibility to addiction.

As discussed previously, we do not know the subcellular mechanism underlying the ethanol-induced increase in α4^∗^ nAChR levels. Because we did not see a change in local mRNA expression, ethanol-induced upregulation, like nicotine-induced upregulation, is a post-translational mechanism. Amongst the possible mechanisms, we believe that two particular post-translational mechanisms are unlikely to occur. First, although an increase in NAc dopamine could augment acetylcholine (ACh) levels, and this could, in turn, lead to desensitisation of α4β2 nAChRs, desensitisation does not appear to cause upregulation (Henderson and Lester, 2015). Second, in neurons, nicotine does not affect the rate of nAChR endocytosis from the plasma membrane (Henderson and Lester, 2015).

Upregulation of nAChRs by chronic exposure to nicotine, at smoking-relevant concentrations, is selective at every level explored to date. Some evidence supports the hypothesis that this selective upregulation is necessary and sufficient for the early stages of nicotine dependence (Miwa et al., 2011; Henderson and Lester, 2015; Henderson et al., 2017). Here we describe these tiers of selectivity. At the level of brain regions, nicotine upregulates nAChRs in hippocampus, cortex, amygdala (Le Foll et al., 2016) and midbrain; but not in the thalamus. Among cell types within brain regions, nicotine upregulates nAChRs strongly in the somata of VTA GABAergic neurons, but only modestly in the somata of VTA dopaminergic neurons. There is also selectivity at the level of somatodendritic vs. axon terminal regions: in dopaminergic neurons, upregulation is modest in the somatodendritic region, but strong in the axon terminals, as also found for binge ethanol in the present study. At the level of nAChR composition, selectivity is also observed: the most consistently upregulated receptors are α4β2^∗^ (where the asterisk denotes the possible presence of other subunits). In some regions, α6β2^∗^ nAChRs are also upregulated. Other nAChR subtypes are upregulated only by much higher, non-pharmacologically relevant nicotine doses. The combinations thought to be resistant to upregulation include α4β4, α4β3, and α7. There is also selectivity at the level of subunit stoichiometry within a nAChR pentamer: (α4)_2_(β2)_3_, but not (α4)_3_(β2)_2_, nAChRs are upregulated by chronic exposure to nicotine. Although no single molecular or molecular level mechanism accounts for these tiers of selectivity, in most cases the upregulated nAChRs are (α4)_2_(β2)_3_. These (α4)_2_(β2)_3_ nAChRs are constitutively retained to some extent in the endoplasmic reticulum (ER) and *cis*-Golgi (Kuryatov et al., 2005; Sallette et al., 2005) and also bind nicotine rather strongly at smoking-relevant doses. Nicotine enters the ER, acts as a stabilising pharmacological chaperone, protects nAChRs against ER-associated degradation (ERAD), and enhances exit of nAChRs from the ER, thus enhancing the number which eventually reach the plasma membrane. This post-translational pharmacological chaperoning is the dominant mechanism of nicotine-mediated upregulation (Henderson and Lester, 2015).

In contrast, a more likely mechanism for ethanol-induced upregulation derives from recent experiments showing that >24 hr exposure to menthol upregulates α4β2^∗^ nAChRs, both in the absence and presence of nicotine. This upregulation may be an example of chemical chaperoning: the menthol binds to as-yet undetermined non-agonist sites, either on the nAChR or on other components of the pathway taken by nAChRs to exit the ER (Henderson et al., 2016, 2017). We suggest that a binge of ethanol acts via a similar mechanism(s). Ethanol increases the open channel state of nAChRs (Wu and Miller, 1994; Forman and Zhou, 1999; Zuo et al., 2004), and this increased stability, if it also occurs in the ER, may protect nAChRs against ERAD. The rather general mechanism of chemical chaperoning should be distinguished from specific pharmacological chaperoning (Kuryatov et al., 2005; Henderson and Lester, 2015), which also occurs within the ER but involves the binding of nicotine or other agonists to nAChRs themselves, at or very near the ACh binding site within the interface between the α4 and β2 subunits.

An identifiable aspect of alcohol use is its strong correlation with nicotine use, and α4^∗^ nAChRs in reward areas of the brain are likely involved in the co-morbidity of these two drugs (Liu et al., 2013), for a comprehensive review see (Tarren and Bartlett, 2017). In line with this, advances in pharmacotherapy for AUDs have indicated the potential of various α4β2^∗^ nAChR agonists such as the smoking cessation drugs cytisine and varenicline in reducing ethanol self-administration (Steensland et al., 2007; Hendrickson et al., 2010; Bito-Onon et al., 2011; Mitchell et al., 2012). Increases in α4^∗^ nAChRs seen after just a single dose of ethanol may then both increase propensity for nicotine use, as well as increase the efficacy of α4^∗^ nAChR targeted therapies for reducing co-morbid alcohol and nicotine use.

## Conclusion

The work presented here not only identifies acute changes to a major population of nAChRs caused by an intoxicating dose of alcohol, but also for the first time visualises specific neuronal populations involved in α4^∗^ nAChR-mediated alcohol behaviours. Whether similar changes occur following a moderate dose of alcohol and/or after chronic exposure, however, was not elucidated in the present work.

If upregulation of α4 containing nAChRs by ethanol does not involve new protein synthesis, it is believed this process may be contingent on post-translational events, and likely has far-reaching implications (Henderson and Lester, 2015). In addition, whether this process impacts long-term alcohol consumption and seeking remains to be investigated.

## Author Contributions

HL provided the α4YFP mice and revised the manuscript. JT performed the WB and qPCR experiments and analysis, and drafted the manuscript. AB performed the IHC experiments and analysis. JT, AB, and SB analyzed and interpreted the results. JT, AB, and SB designed the experiments and revised the manuscript. All authors approved the final version of the manuscript.

## Conflict of Interest Statement

The authors declare that the research was conducted in the absence of any commercial or financial relationships that could be construed as a potential conflict of interest.

## Supplementary Material

**FIGURE S1 |** Tyrosine hydroxylase expression following acute ethanol exposure in the nucleus accumbens and amygdala. **(a,b)** Quantification of TH levels by Western Blot analysis shows no effect of ethanol in the NAc **(a)** or the Amg **(b)**. Data are presented as mean k counts for TH expressed as a percentage of GAPDH k counts ± SEM (two-tailed unpaired *t*-test, **a**: NAc: *p* = 0.30, *n* = 4; and **b**: Amg: *p* = 0.55, *n* = 7–8). **(c,d)** Quantitative immunohistochemistry confirmed the absence of change in TH expression in the NAc **(c)** and the Amg **(d)**. Data are presented as mean volumetric density of fibres in μm^3^ per 10^3^ μm^3^ of tissue (two-tailed unpaired *t*-test, **c**: NAc: *p* = 0.62; and **d**: Amg: *p* = 0.99, compared to saline).

**FIGURE S2 |** Immunohistochemistry against TH labels more the dopaminergic neurons and terminals than the noradrenergic neurons and terminals. **(a,b)** Immunolabelling of TH-immunoreactive dopamine (DA) neurons in the ventral tegmental area (VTA, **a**) and TH-immunoreactive noradrenergic (NA) neurons in the locus coeruleus (LC, **b**). **(c,d)** Immunolabelling of TH-immunoreactive terminals in DA terminal-rich region, the NAc **(c)** and NA terminal-rich region, the paraventricular nucleus (PVN, **d**). Scale bar: 50 μm.

## Abbreviations

Amg: amygdala
AUDs: alcohol use disorders
BEC: blood ethanol concentration
BLA: basolateral amygdala
CeA: central amygdala
GAPDH: glyceraldehyde 3-phosphate dehydrogenase
GFP: green fluorescent protein
HPA: hypothalamic-pituitary-axis
HPRT: hypoxanthine-guanine phosphoribosyltransferase
LA: lateral amygdala
LORR: Loss of righting reflex
NAc: nucleus accumbens
nAChR: nicotinic acetylcholine receptor
PFC: prefrontal cortex
qRT-PCR: quantitative reverse transcription polymerase chain reaction
RORR: return of righting reflex
TH: Tyrosine hydroxylase
VTA: ventral tegmental area
YFP: yellow fluorescent protein
