# Supplementary figures and images for: Acute Ethanol Administration Upregulates Synaptic α4-Subunit of Neuronal Nicotinic Acetylcholine Receptors within the Nucleus Accumbens and Amygdala
Source: Front Mol Neurosci. 2017 Oct 24;10:338. doi: 10.3389/fnmol.2017.00338 (PMC5660714; doi:10.3389/fnmol.2017.00338)

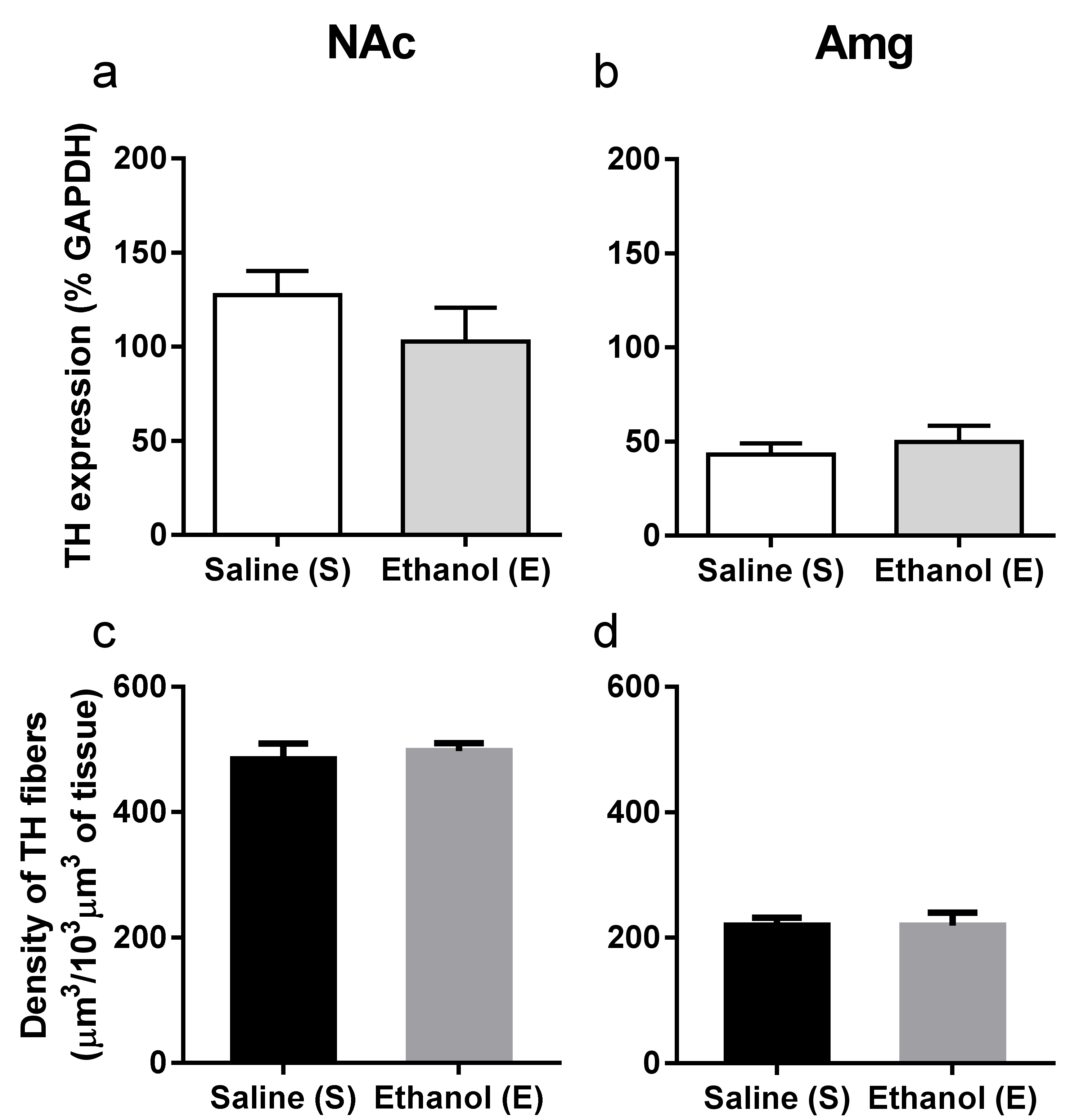

Supplement: Supplementary file 1 [file Image_1.TIF]

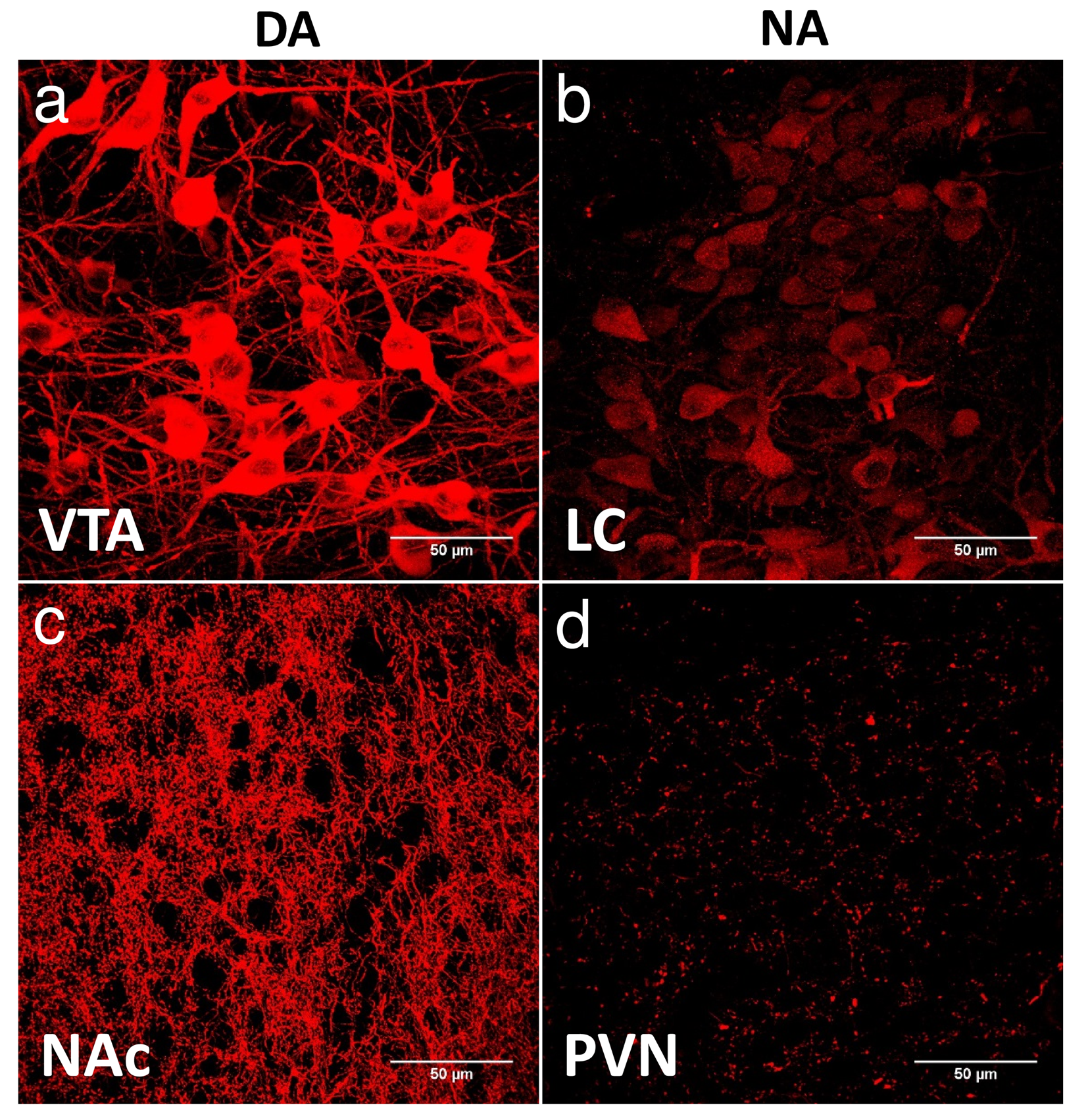

Supplement: Supplementary file 2 [file Image_2.TIF]
